# Oleum Gaultheria—Oil of Wintergreen

**Published:** 1883-04

**Authors:** E. S. Bates

**Affiliations:** Professor of Therapeutics and Materia-Medica, Columbia Veterinary College


### Art. XVI.—OLEUM GAULTHERIA—OIL OF WINTERGREEN.

BY E. S. BATES, M.D.,
*Professor of Therapeutics and Materia-Medica, Columbia Veterinary College.*

UNTIL quite recently this drug has been known only as an aromatic remedy possessing slight tonic properties.

It is an indigenous plant, common in the Northern and Middle States, and chiefly known as checkerberry plant, wintergreen, partridgeberry, deerberry, teaberry, mountain tonic.

The medicinal virtues of the plant reside in a volatile oil known as Oleum Gaultheria, obtained by distillation. When first prepared it is nearly colorless, but age gives it a brownish-yellow or red color.

It has a sweetish, slightly pungent, peculiar taste, and an agreeable characteristic odor.

It is an unusually heavy oil, being the heaviest of the known essential oils.

It is a methylic ether of salicylic acid, containing 90 per cent. of the methysalicylic acid and 10 per cent. of a peculiar substance known as gaultherilen, a carbo-hydrogen which probably belongs to the turpenes.

It dissolves readily in all proportions with alcohol and ether, and to some considerable extent is soluble in water.

When administered internally it is quickly absorbed into the blood and rapidly eliminated chiefly through the kidneys imparting its own odor to the urine.

It is eliminated *very rapidly*, its presence being very perceptible in the urine within an hour after its exhibition.

*Therapeutics.*—Until quite recently this drug has been used only for its aromatic and placeboic properties. Within a short time it has come to be considered one of the most efficient drugs for the cure of acute rheumatism. Under its exhibition the pyrexia of that disease is very efficiently controlled.

The joint pains quickly disappear, and in the general influence it has over the various morbid symptoms of this painful affection it seems to be the equal, if not the superior, of any of the salicylic compounds,

It has proved equally as efficacious in the treatment of the sub-acute form as in the acute.

It manifests very decided antiseptic properties which are of practical importance.

One part of the oil to 200 parts of urine will preserve it nearly three weeks.

Urine voided one hour after its exhibition remains free from decomposition twelve days,

Ordinary urine decomposes quickly, and very unpleasant is the odor arising from it.

In active practice it is not always convenient to make a careful examination of a given specimen just when the examination would be least offensive and most satisfactory.

A small proportion of this drug mixed with a given specimen will preserve it sufficiently long without in any way interfering with a practical examination.

It is agreeable to the taste, and what is also of importance in our practice when such large quantities are required in treating larger animals, it is comparatively cheap. Its cheapness does not, however, prevent it from being deteriorated by adulterations with oil of turpentine, oil of birch, and also diluted with alcohol.

The dose for a horse varies from one to two drachms, It should be exhibited in frequently repeated doses. Being very rapidly eliminated its effects are of comparatively temporary duration.

The best results are, however, obtained by giving the drug in increasing doses at short intervals until the chief symptoms are brought under control, and then in gradually decreasing doses continue its exhibition for six or seven days after all traces of the disease have disappeared.

The mode of administration varies. It can be given mechanically mixed with oil or milk.

The best method is to rub it up with the carbonate of magnesia, one drachm of the oil to the half ounce of magnesia, and give it in the form of a capsule or wrapped up in wafers.

The crudest mode is to shake it up well with water, and exhibit as a drench.

